# Orotracheal Intubation Challenges in an Anticipated Difficult Mask Ventilation, Preoxygenation, Tricky Laryngoscopy, and Supraglottic Airway Device Insertion: A Case Report

**DOI:** 10.7759/cureus.70461

**Published:** 2024-09-29

**Authors:** Bheemas Atlapure, Habib Md R Karim, Baby Pegu, Ankita Medhi

**Affiliations:** 1 Anaesthesiology, Critical Care, and Pain Medicine, All India Institute of Medical Sciences, Guwahati, Assam, IND

**Keywords:** airway management, fibreoptic bronchoscope, i-gel, orotracheal intubation, predicted difficult airway

## Abstract

With the advancement of technology, equipment, and airway management knowledge, anticipated difficult airway (DA) management has come a long way towards excellence. Usually, anticipated difficulties are related to bag-mask ventilation (BMV), laryngoscopy, intubation, or supraglottic airway placement; all in a single patient pose exceptionally challenging airway management. We may electively plan a surgical airway, but the option may not be available, especially when the patient provides tracheostomy permission only for emergency airway management, not for an elective. A 48-year-old male patient with a probable diagnosis of midline lethal granuloma presented with right-side nasal blockade, deformity, and near-total blockade of the left nasal cavity with right-sided mid-facial swelling, pain, and foul-smelling discharge and an ulcerated hard palate and was scheduled for an endoscopic biopsy. The airway examination predicted difficult bag-mask ventilation, pre-intubation oxygenation, risky laryngoscopy, and supraglottic airway insertion. Even airway topicalization, sedation, and preparation for awake intubation were challenging. Resource limitations and unexpected desaturation while attempting awake intubation led to an emergent situation; i-gel came as a rescue, and ultimately, the definitive airway was secured using a 6.5 mm cuffed endotracheal tube (ETT), railroaded over a fibreoptic bronchoscope (FOB), and inserted through i-gel. We present the case to highlight the challenges and discuss the possible remedies where our technique can be an alternative for cases with difficult mask ventilation, intubation, and supraglottic airway insertion.

## Introduction

Difficult airway (DA) management is one of the most critical challenges for anaesthesiologists during their daily clinical practice. In patients with predicted difficult airways, fibreoptic bronchoscope (FOB)-guided intubation, while the patient is spontaneously breathing, is the criterion standard technique for securing the airway [1]. Latest-generation equipment like video laryngoscopes (VLs) and supraglottic airway devices (SADs) developed over the last decades has revolutionized airway management and allowed orotracheal intubation (OTI) through them [2-4]. SADs also ease manoeuvrability with the flexible FOB, achieving high OTI success rates [4]. The i-gel (Intersurgical Ltd., Wokingham, Berkshire, United Kingdom), an SAD, has also been used in DA management [5]. The device is well tolerated by awake or lightly sedated patients with spontaneous breathing [6,7]. However, literature is scarce on OTI using FOB guidance through i-gel during emergent airway management under sedation in patients with predicted bag-mask ventilation (BMV), difficulty with inefficient pre-oxygenation, difficult intubation, risky direct laryngoscopy (DL), and SAD insertion. We discuss a case highlighting the challenges and possible remedies in such a scenario where our employed technique can be an alternative for cases with difficult BMV, intubation, and SAD insertion.

## Case presentation

We obtained written permission from the patient to present the case. A 48-year-old male, 73 kg, 170 cm (body mass index 25.26 kg/m^2^) with a probable diagnosis of midline lethal granuloma presented with right-side complete nasal blockade, deformity, and near-total blockade of the left nasal cavity with right-sided mid-facial swelling, pain, and foul-smelling discharge. He also had an ulcerated hard palate, which was likely an extension of the mass. A previous biopsy attempt under local anaesthesia was unproductive. The mass was rapidly growing over the week and was scheduled for an endoscopic biopsy. He was also a known case of hepatitis B and C seropositivity and consumed tobacco and alcohol, which he had stopped recently. However, he was living a healthy life without any limitations or symptoms until before a month; there was also no history of any other comorbidities in past illnesses.

**Figure 1:**
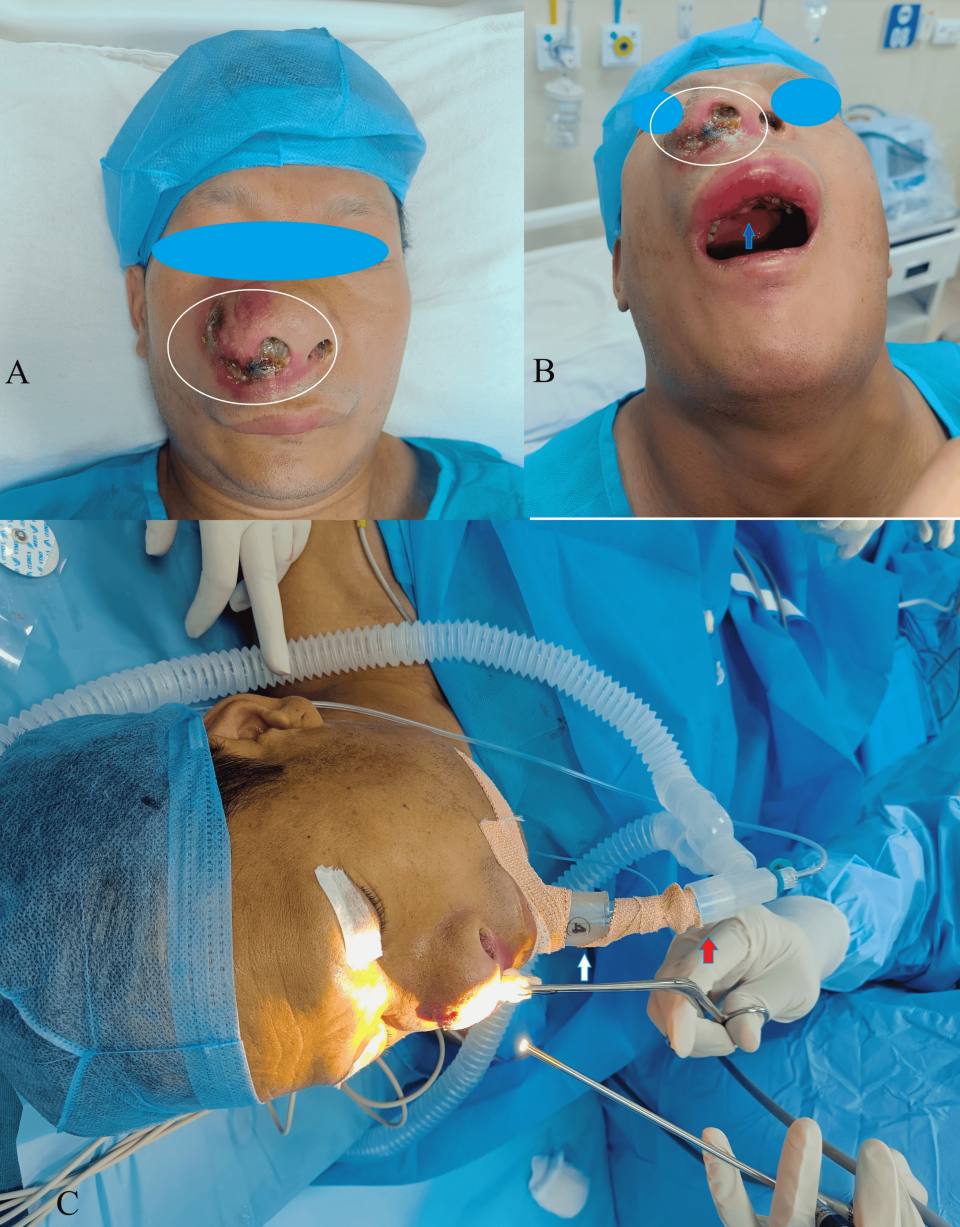
Clinical gross images showing the mass (encircled) in (A) and (B) and hard palate ulcer and growth (arrow) in (B) and endotracheal tube fixed along with i-gel and connected to closed circuit in (C).

Airway assessment revealed a modified Mallampati grade II, mouth opening of 4 cm, thyromental distance of 6.5 cm, upper lip bite test grade III, and normal neck mobility. On local examination of the nose, the patient had diffuse swelling and redness over the dorsum of the nose on the right side with crusting and foul-smelling discharge in the tip of the nose and bilateral nostril, whitish necrotic slough with red polypoidal mass inside the nasal cavity (Figure 1A). An oral examination also revealed multiple loose teeth in the upper jaw (incisors on either side and canine and pre-molars of the right side) associated with ulceration and slough in the rigid palate (Figure 1B). A contrast-enhanced computed tomography scan showed a sizeable iso-dense lesion of 72 mm × 36 mm and minimal enhancement in the right maxillary sinus and nasal cavity. The right osteo-meatal complex was obstructed by the lesion, with bony remodelling seen in the right nasal cavity and mucosal thickening seen in the right ethmoidal sinus. The anterior nares of the right nostril were obstructed by the swelling of the right nostril with a sessile polyp in the left maxillary sinus and mild mucosal thickening in the right frontal sinus. The histopathology examination report of the biopsy of the anterior hard palate showed severe dysplasia with extensive necrosis. The chest X-ray appeared within normal limits.

The above findings indicated difficult BMV, preoxygenation, challenging DL, and difficult SAD insertion despite a suitable oropharyngeal inlet. The patient did not consent to an elective surgical airway to worsen the scenario. Under standard monitoring and keeping the DA cart ready, we supplemented O_2_ through the circuit, just keeping the mask above the oronasal structures and asking the patient to take breaths through the mouth for five minutes. An awake FOB-guided OTI was deemed unsuitable owing to poor airway preparation, loose teeth negating the use of bite blocks, and possible trauma and bleeding in case the patient turns out non-cooperative or coughs and bucks. So, we planned to proceed with DL under mild sedation and either proceed to OTI if the laryngeal inlet is well visualized (plan A) or immediately put an i-gel under DL, avoiding a touch to the ulcerated palatal growth (plan B).

Nevertheless, FOB-guided OTI through the SAD under conscious sedation was the alternative plan (plan C), and emergent front of neck access (FONA) was a life-saving rescue airway management plan (plan D). The patient was premedicated with intravenous glycopyrrolate 0.2 mg, fentanyl 100 mcg, and propofol 0.5 mg/kg (35 mg) slow in running drip-set and maintained conscious sedation. While still responding to verbal stimuli, a check DL was done by an experienced anaesthesiologist, which showed a Cormack Lehane (C&L) grade IV, and OTI was deemed not feasible. Meanwhile, the patient coughed and went to breath-hold, and desaturation started. Considering the difficult BMV, we inserted i-gel size-4 using the DL as the hard palate had necrosis and slough. We could achieve good ventilation and SpO_2_; however, the patient ended up with deep sedation to make i-gel tolerable, which was achieved using sevoflurane and propofol. As a secured airway was needed for surgery, we tried to intubate the trachea using a 6.5 mm cuffed endotracheal tube (ETT) through the i-gel as a conduit.

However, we were unsuccessful, and ETT could not be negotiated beyond the i-gel bowl. Therefore, we improvised the plan to introduce the ETT to the bowl of i-gel, insert them under DL, perform FOB through ETT, and railroad ETT for intubation. An adult FOB (Olympus Medical Systems Corp., Tokyo, Japan) was used, and the same ETT tube was positioned, confirmed under indirect vision, and by 5-point auscultation and capnography reading. After proper ET positioning, Inj. propofol 50 mg and vecuronium bromide 7 mg were administered. The ETT adaptor was secured with tape to the in-situ i-gel and the ETT, as we did not have the tube pusher at that moment (Figure 1C).

Maintenance of anaesthesia and analgesia after that followed the usual daily practice of our institute. The surgery went on smoothly for 90 minutes. After completion of the surgical procedure and haemostasis, nasal packing was done, following which the patient was planned for extubation. Following the neuromuscular blockade reversal, the ETT cuff was deflated and removed once the patient had adequate spontaneous, regular breathing with minimal pressure support of 6 cm H_2_O. Still, the i-gel was left in situ until the patient regained full consciousness, given the patient’s difficult airway. The patient was observed in the post-anaesthesia care unit for an hour and then discharged to the ward.

## Discussion

The present case had many challenges, but we could overcome them using different techniques. Although our plan B, using the i-gel placement, was successful, the further airway management did not go as planned as the patient ended up with (?) laryngospasm and intolerance to i-gel. The FOB from plan C came to the rescue but was not in the way planned and required some tailoring according to the situation and resource limitations. Retrospectively, if we introspect, we can argue that awake FOB through the oral route should have been performed or elective tracheostomy should have been done. However, these were not without hurdles, as the patient party did not consent to elective tracheostomy.

Further, Mallampatti Grade, mouth opening, thyromental distance, sterno-mental distance, and neck movements were favourable awake airway management trials and proceeded with elective tracheostomy. However, awake tracheal intubation requires preoxygenation, oxygen supplementation during airway management, local anaesthesia topicalization, and cautious mild sedation [8]. Unfortunately, all these were not feasible or effective in our case due to the pathology involving the upper airway. Further, awake FOB through the oral route needs the patient’s full cooperation and bite block. It was considered risky because the patient had an ulcerated hard plate and multiple loose teeth. Any injury to the upper teeth or hard plate could lead to bleeding and jeopardize the FOB procedure [9].

Considering the above facts, our plan A was to perform a check DL under mild sedation, which can again be argued to be performed using a VL. Unfortunately, our institute (still in the early construction phase) was still procuring VLs. Therefore, we had to perform the check DL using a conventional Macintosh blade, which usually requires a significantly higher force [10]. Hence, minimal analgo-sedation while maintaining consciousness and spontaneous breathing was the primary plan to find the C&L grade and OTI feasibility. Unfortunately, the patient did not even tolerate the check DL and went to hold his breath (? laryngospasm), requiring immediate intervention. Desaturation further led to an emergency. As OTI was deemed not feasible with C&L grade IV, we only had two options: SAD insertion and trying positive pressure to break the breath-hold or front-of-neck access (FONA). While preparing for FONA, SAD (i-gel), Larsen's manoeuvre, and inhalational sedation with sevoflurane worked. Nevertheless, the rapid desaturation of the patient was very surprising, especially when O2 supplementation was done for five minutes.

SADs play multiple roles in the management of DA and are indicated for DA rescue in cases of impossible intubation and/or inadequate manual ventilation. SADs can achieve adequate ventilation in difficult intubation situations, especially in cases of unanticipated DA [11-13]. When a patient is anticipated to have DA and has a predictor of difficult or risky SGA insertion, the scenario becomes complex.

Tracheal intubation is the safest airway maintenance method during GA, but it is impossible in 0.1-0.4% of patients [14]. While initial attempts to secure the airway were unsuccessful, it was achieved via FOB-guided tracheal intubation via the i-gel™ lumen. The Difficult Airway Society guidelines for the unanticipated DA recommend that, after failed intubation, the primary goal should be maintaining and restoring alveolar oxygenation via the insertion of a second-generation SAD. Successful SAD insertion and reoxygenation provide practitioners time to “stop and think” and assess their options [13,15]. Intubation via a SAD is one of the several options, but only when the situation is deemed appropriate [15]. Although an overall success rate of 95.7% has been reported in a series of 1100 patients using a blind technique, blind intubation attempts via a SAD are, however, not recommended [13,16]. The first-attempt success rates are higher using FOB guidance, and a guided technique benefits patients with difficult airways. Success rates for fibreoptic tracheal intubation via the i-gel™ are equivalent to the Laryngeal Mask Airway (LMA®) Fastrach™ (Teleflex®, Athlone, Westmeath, Ireland) [17,18].

We used the i-gelTM size 4, which can pass size 7-mm cuffed endotracheal tube (ETT). However, we introduced a slightly smaller cuffed 6.5 mm ETT by railroading technique using the adult FOB to minimize factors that might impede first-pass intubation success, such as excessive friction when passing the ETT through the i-gelTM lumen or ETT impingement on laryngeal structures due to the size difference between the bronchoscope and a larger ETT [15]. DA management algorithms recommend obtaining a secure airway to ventilate the patient before inducing apnea. With an adequate i-gel mask insertion in spontaneous ventilation, a possible “cannot ventilate” scenario was solved, and we could approach subsequent OTI from a nonemergency pathway [11,13].

An adequate level of sedation and the appropriate administration of local oropharyngeal anaesthesia is crucial for performing any OTI technique for an “awake” patient, whether it is with a SAD, with a FOB, or with a VL [13]. The proper combination of both methods facilitates the work of the anaesthesiologist, provides comfort to the patient, and avoids adverse events due to reflexes such as coughing, laryngospasm, or airway injuries [13,19]. A recent case report also indicates using i-gel to rescue DA following an attempt to secure the airway using FOB and VL, and subsequently, the airway was secured by FOB through i-gel [15]. FOB-guided intubation via an i-gel may negate the need for neuromuscular blockade if situation-specific concerns exist and have benefits due to its structural characteristics. Our case modified the technique by keeping the i-gel in situ with the ETT throughout the procedure, which also indirectly worked as throat packing, protecting the laryngeal inlet. Following surgery and neuromuscular blockade reversal, ETT was removed initially, followed by FOB through the i-gel to confirm no secretions around the vocal cord. The i-gel was removed at last once the patient was completely awake.

## Conclusions

The case demonstrates an effective method of securing a predicted difficult airway using i-gel under moderate sedation and subsequent use as a conduit to introduce a fibreoptic bronchoscope to perform OTI using cuffed ETT. However, such airway management might result in an adverse situation needing an immediate, tailored approach over and above pre-procedure planning, including a slight deviation from the plan. Airway rescue using FOB-guided OTI via the i-gel lumen where initial intubation attempts have failed might be a life-saving technique in an emergently difficult airway. Postoperatively, i-gel can even be safely kept in the patient's upper airway until the patient becomes fully awake.
